# The Role of Eustachian Tube Dysfunction in Recurrent Chronic Otitis Media: A Cross-Sectional Study of Anatomical and Functional Variations

**DOI:** 10.3390/healthcare13010077

**Published:** 2025-01-03

**Authors:** Sarah Alshehri, Abdullah Musleh

**Affiliations:** Otology and Neurotology, Department of Surgery, College of Medicine, King Khalid University, Abha 61423, Saudi Arabia; amusleh@kku.edu.sa

**Keywords:** chronic otitis media, Eustachian tube, tympanometry, anatomical variations, functional impairments

## Abstract

**Background/Objectives**: Recurrent chronic otitis media (rCOM) is a major cause of hearing impairment, often linked to Eustachian tube (ET) dysfunction. Anatomical abnormalities, such as ET narrowing and obstructions, and functional impairments, including poor pressure regulation, play significant roles in rCOM recurrence. This study aimed to (1) identify anatomical variations of the ET in patients with rCOM using high-resolution imaging; (2) evaluate ET functional status using tympanometry, tubomanometry, and ET function tests; and (3) assess the correlation between anatomical variations and functional impairments in predicting rCOM recurrence. **Methods**: A cross-sectional study was conducted on 212 patients with rCOM and 212 controls. High-resolution CT and MRI were used to assess ET anatomy, while functional status was evaluated using tympanometry, tubomanometry, and Valsalva maneuver tests. Statistical analyses, including *t*-tests, Pearson correlation, and Cox proportional hazards models, were applied to examine the relationship between anatomical and functional impairments and rCOM recurrence. **Results**: Significant anatomical differences were observed in the rCOM group, including ET narrowing (24.53% vs. 11.32%, *p* = 0.014) and curvature (32.08% vs. 14.15%, *p* < 0.001). Functional impairments were also more prominent in rCOM patients, with higher ET opening pressure (120.56 ± 14.34 dPa vs. 85.78 ± 12.98 dPa, *p* < 0.001) and lower Valsalva maneuver success rates (62.32% vs. 89.56%, *p* < 0.001). Cox regression indicated that anatomical and functional impairments significantly predicted faster recurrence (HR for tympanometry peak pressure = 1.56, *p* < 0.001). **Conclusions**: The ETs anatomical and functional impairments are significant predictors of rCOM recurrence. A combined assessment of these factors can improve diagnostic accuracy and guide more targeted interventions to prevent recurrence.

## 1. Introduction

Recurrent chronic otitis media (rCOM) remains a significant clinical challenge due to its persistent nature and associated complications, such as hearing loss and structural damage to the middle ear [1]. While previous research has highlighted various risk factors for rCOM, the precise contributions of Eustachian tube (ET) dysfunction remain underexplored [2]. This study seeks to address the research question: “How do anatomical and functional impairments of the Eustachian tube contribute to the recurrence of chronic otitis media, and what is the interplay between these factors in predicting recurrence rates?” Specifically, we hypothesize that (1) anatomical variations of the ET, including narrowing, curvature, and obstructions, are significantly associated with rCOM recurrence; (2) functional impairments, such as reduced middle ear compliance and abnormal ET opening pressures, exacerbate disease recurrence; and (3) a combined assessment of these factors improves diagnostic accuracy and recurrence prediction. By integrating advanced imaging techniques and functional tests, this study aims to fill a critical gap in understanding the relationship between ET dysfunction and rCOM recurrence.

Chronic otitis media (COM) is a persistent inflammation of the middle ear and is a major cause of hearing impairment, particularly in children and adults with recurrent infections [1]. rCOM, characterized by multiple episodes of middle ear infections over time, poses significant clinical challenges due to its tendency to cause long-term structural damage, including tympanic membrane perforation, ossicular erosion, and the development of cholesteatoma [2]. Despite advancements in medical and surgical management, many patients continue to experience repeated episodes of COM, leading to a high risk of hearing loss, reduced quality of life, and increased healthcare costs [3]. Understanding the underlying mechanisms contributing to rCOM is essential to improving the diagnostic and therapeutic strategies that can prevent recurrence and mitigate its complications [4].

One of the key factors implicated in the pathophysiology of rCOM is the dysfunction of the Eustachian tube (ET), which plays a critical role in middle ear ventilation, pressure regulation, and drainage of secretions [2]. Anatomical variations in the ET, such as narrowing, abnormal angulation, or the presence of structural obstructions, have been identified as potential contributors to the development and recurrence of COM [5]. High-resolution imaging techniques, such as CT and magnetic resonance imaging (MRI), have enabled a detailed examination of ET anatomy in patients with rCOM, allowing for the identification of specific structural abnormalities that may predispose individuals to recurrent infections [6]. These imaging modalities provide critical insights into how anatomical variations may impair ET function, potentially leading to persistent middle ear inflammation and infection [6]. Studies have suggested that patients with narrower or abnormally angled ETs are more prone to rCOM, but further investigation is needed to fully understand the extent to which these variations contribute to disease recurrence [7].

In addition to anatomical abnormalities, functional impairments of the ET are considered major determinants of middle ear pathology [8]. Evaluating the functional status of the ET in patients with rCOM is crucial to understanding how its dysfunction contributes to disease recurrence [9]. Objective assessments, including tympanometry, tubomanometry, and other ET function tests, offer valuable information about the pressure-regulation capabilities of the ET [10]. Tympanometry assesses middle ear compliance and pressure, helping to identify negative pressure conditions that may indicate ET dysfunction [11]. Tubomanometry evaluates the ability of the ET to open and close properly, particularly during swallowing or pressure-equalizing maneuvers like the Valsalva maneuver [12]. These functional tests can help detect subtle abnormalities that may not be visible on imaging but still significantly impact the ETs ability to maintain normal middle ear physiology [12]. Studies have demonstrated that patients with rCOM often exhibit abnormal tympanometry findings and impaired ET opening pressures, further highlighting the role of functional dysfunction in recurrent infections [13,14].

Given the significant impact of both anatomical and functional impairments on ET dysfunction, it is essential to assess the correlation between these two factors to better understand their combined influence on rCOM recurrence [15]. Anatomical variations, such as ET narrowing or abnormal positioning, may exacerbate functional impairments by further restricting the ETs ability to open and equalize pressure [16,17]. Conversely, functional impairments may worsen anatomical issues, creating a vicious cycle of middle ear dysfunction that promotes chronic inflammation and infection [18]. Evaluating the interaction between anatomical and functional impairments is critical to identifying high-risk patients and developing targeted interventions that can prevent rCOM recurrence [19]. Despite previous studies exploring the individual contributions of anatomical and functional ET dysfunction, few studies have comprehensively examined the relationship between these factors in predicting recurrence rates of COM [20].

This study is needed because of the current gap in understanding the combined effects of anatomical and functional ET dysfunction on the recurrence of COM [21]. Previous research has primarily focused on either anatomical or functional aspects in isolation without considering how these factors may interact to influence the likelihood of disease recurrence [1,22]. Additionally, while high-resolution imaging and objective functional assessments have been utilized separately in clinical practice, there is limited data on the predictive value of combining these modalities to assess rCOM risk [23]. Addressing this research gap is critical to advancing diagnostic accuracy and improving patient outcomes through more personalized and targeted treatment strategies. Furthermore, while surgical interventions, such as tympanostomy tube placement, are often used to manage rCOM, they do not address the underlying ET dysfunction [24], and many patients continue to experience recurrent infections post-surgery. Understanding the anatomical and functional contributions to ET dysfunction may lead to better long-term management strategies that reduce reliance on surgical interventions and improve overall disease outcomes [25].

The present study aims to investigate the anatomical and functional variations of the Eustachian tube in patients with rCOM using high-resolution imaging techniques and objective functional assessments. The first objective is to identify anatomical variations of the ET in patients with rCOM through high-resolution CT and MRI, focusing on specific structural abnormalities such as ET narrowing, abnormal angulation, and obstructions. The second objective is to evaluate the functional status of the ET in these patients using tympanometry, tubomanometry, and ET function tests, assessing their ability to maintain middle ear pressure and equalize during pressure changes. The third objective is to assess the correlation between anatomical variations and functional impairments in predicting recurrence rates of COM.

## 2. Materials and Methods

### 2.1. Design

This cross-sectional study was conducted between April 2023 and February 2024 at the Otolaryngology tertiary care hospital specializing in ear, nose, and throat disorders. The hospital is equipped with advanced imaging technologies, including high-resolution CT and MRI, and has a dedicated team of otologists and radiologists experienced in Eustachian tube dysfunction and middle ear pathologies. The study involved 212 patients diagnosed with rCOM and 212 control participants. High-resolution imaging techniques, including CT and MRI, were employed to evaluate anatomical variations of the Eustachian tube, while functional assessments were conducted using tympanometry, tubomanometry, and Eustachian tube function tests. All participants provided written informed consent prior to their inclusion in the study. Ethical approval was obtained from the hospital’s institutional review board (REC#456-2023) on 22 March 2023, and the research adhered to the principles of the Declaration of Helsinki. (CT (Siemens SOMATOM Force, 0.5 mm slice thickness, Siemens Healthcare, Erlangen, Germany)).

### 2.2. Participants

A total of 424 participants were enrolled in this cross-sectional study, comprising 212 patients diagnosed with rCOM and 212 healthy controls. Participants were recruited from the Otolaryngology Department of a tertiary hospital in Aseer between April 2023 and March 2024. Patients met clinical criteria for rCOM, including a documented history of at least three otitis media episodes within the prior year, persistent middle ear effusion, or recurrent tympanic membrane perforations lasting over three months despite appropriate treatment. Diagnoses were confirmed through otoscopic examination, audiometry, and tympanometric evaluation.

Inclusion criteria for the rCOM group required participants to be 18 to 65 years old, with no prior major ear surgeries, such as mastoidectomy, that could alter Eustachian tube function. Control group participants were selected from the same age range and confirmed to have no history of otitis media, middle ear effusion, or Eustachian tube dysfunction, as verified through normal tympanometry and otoscopy. Both groups were screened to exclude individuals with active upper respiratory infections, congenital ear abnormalities, head and neck tumors, or previous Eustachian tube surgeries, such as balloon dilation or tympanostomy tube placement. Additional exclusion criteria included ongoing nasal polyps, chronic sinusitis, or allergies requiring treatment, as well as immunocompromising conditions like autoimmune or chronic systemic diseases. Patients with active tympanic membrane perforations were excluded from tympanometry to ensure the validity of functional assessments.

Participants were selected using a combination of clinical screening and convenience sampling. Controls were drawn from hospital staff, healthy volunteers, and individuals attending routine checkups for non-otological conditions. Matching by age, gender, and body mass index (BMI) was performed to ensure demographic comparability between groups, minimizing potential confounding. Further confounding variables, such as smoking status, occupational noise exposure, air pollution, and prior respiratory allergies, were recorded and controlled for during statistical analyses to ensure robust findings. All participants provided informed consent and underwent standardized assessments, including high-resolution imaging (CT and MRI) and functional Eustachian tube tests, to evaluate both anatomical and functional characteristics. This systematic selection process ensured the inclusion of a well-defined population for a comprehensive investigation of the role of Eustachian tube dysfunction in rCOM recurrence.

### 2.3. Variables

The primary outcome variable in this study was the recurrence of COM, measured by the frequency of otitis media episodes within the past year, confirmed by clinical history and medical records. The recurrence rate was categorized based on the number of episodes per year and was further stratified into low-frequency (less than three episodes) and high-frequency (three or more episodes) recurrence. Secondary outcomes included the anatomical and functional characteristics of the ET, which were assessed through high-resolution imaging and functional tests.

Anatomical variables of the Eustachian tube were evaluated using computed tomography (CT) and MRI [26]. The variables measured included ET angle, length, width, presence of structural abnormalities such as narrowing, curvature, and obstructions, and the position of the ET relative to surrounding structures, including the nasopharynx and middle ear [27]. Measurements of ET angle (degrees), length (millimeters), and width (millimeters) were taken using standardized radiological techniques, while the presence of structural anomalies was documented based on visual assessment by experienced radiologists [27]. The degree of ET narrowing, curvature, and abnormal positioning was quantified in terms of percentages of affected patients in the COM and control groups [27]. Functional variables were assessed through tympanometry, tubomanometry, and Eustachian tube function tests, including the Valsalva maneuver [28]. Tympanometry provided data on middle ear compliance and peak pressure, measured in decapascals (dPa), with negative peak pressure values indicating ET dysfunction [29]. Tubomanometry measured ET opening pressure (dPa), reflecting the ETs ability to open during swallowing or pressure equalization maneuvers [29]. The success of the Valsalva maneuver expressed as a percentage, was used to assess the ETs ability to equalize pressure [29]. These functional variables were measured using standardized equipment, including tympanometers and tubomanometers, and were recorded as continuous variables.

High-resolution CT scans (Siemens SOMATOM Force, 0.5 mm slice thickness) and MRI (Philips Achieva 3T, T2-weighted, and CISS sequences) were used to evaluate ET anatomy, including angle, length, width, and structural abnormalities such as narrowing, curvature, and obstructions [30]. Imaging was analyzed by two trained radiologists blinded to group assignment, ensuring unbiased interpretation. Radiologists underwent standardized training for ET measurements, and inter-observer reliability was confirmed with intraclass correlation coefficients (ICCs) exceeding 0.90. Functional assessments included tympanometry (GSI TympStar Pro) to measure middle ear compliance and peak pressure, with thresholds of <−100 dPa or compliance < 0.3 cm^3^ indicating dysfunction [30]. Tubomanometry (Micromedical FL900) assessed ET opening pressure, considering values > 100 dPa as impaired [31]. The Valsalva maneuver was evaluated for success in equalizing middle ear pressure, verified by post-maneuver tympanometric measurements [31]. All tests and imaging protocols were performed by trained personnel following standardized procedures, with equipment calibrated per manufacturer guidelines to ensure precision and reliability.

Demographic and clinical variables, such as age, gender, body mass index (BMI), smoking status, and history of allergies or previous ear surgeries, were also collected through patient interviews and medical records. These variables were included to assess potential confounders and adjust for factors that may influence ET function and the recurrence of COM. Additionally, smoking status was categorized as current smoker, former smoker, or non-smoker, and history of allergy was recorded as a binary variable (yes/no).

### 2.4. Sample Size Calculation

The sample size calculation for this study was performed using G*Power software version 3.1 (Heinrich-Heine-Universität Düsseldorf, Düsseldorf, Germany) to ensure sufficient power to detect significant differences and correlations between anatomical and functional variations of the ET in patients with rCOM. A moderate effect size (Cohen’s d = 0.3) was anticipated based on the Zhen et al. [32] study investigating ET dysfunction in otitis media [32], with a power of 0.80 and an alpha level of 0.05. Using these parameters, it was determined that a minimum of 191 participants would be required to detect meaningful relationships between the variables under investigation. To account for potential data loss or incomplete assessments, a dropout rate of 10% was factored into the calculation, increasing the target sample size to 212 participants per group.

### 2.5. Data Analysis

Data analysis was conducted using SPSS version 24, with statistical significance set at *p* < 0.05. Descriptive statistics summarized demographic and clinical characteristics, presenting continuous variables as mean ± standard deviation (SD) and categorical variables as frequencies (percentages). Independent *t*-tests assessed group-level differences in continuous variables, such as Eustachian tube (ET) angle, length, width, and tympanometry peak pressure, between the rCOM and control groups. Cohen’s d was calculated to quantify effect sizes, highlighting the magnitude of these differences. Multivariate analyses, including multiple linear regression models, were employed to address confounding variables, such as age, gender, smoking status, allergy history, and prior upper respiratory infections. These models adjusted for potential confounders when evaluating the relationships between anatomical and functional ET impairments and COM recurrence. Subgroup analyses stratified patients by recurrence frequency and severity, with comparisons made using independent *t*-tests and analysis of variance (ANOVA). Pearson correlation coefficients were computed to determine the strength and direction of associations between anatomical and functional variables and COM recurrence rates. Interaction terms in multiple regression models explored the combined effects of anatomical and functional impairments, such as ET angle and tympanometry peak pressure. Cox proportional hazard models were utilized to estimate the time to COM recurrence, with hazard ratios (HR) and 95% confidence intervals (CI) quantifying the influence of key predictors. Kaplan-Meier survival curves visualized the time-to-recurrence data, providing a detailed assessment of the impact of ET dysfunction.

## 3. Results

Patients in the COM group had a significantly higher proportion of smokers, more previous episodes of COM, and greater impairments in Eustachian tube function (ETD test) compared to the control group, with all *p*-values < 0.05 (Table 1).

Additionally, the COM group demonstrated more abnormal tympanometry and tubomanometry results, along with a higher prevalence of allergy history and previous surgeries. However, no significant differences were observed between the two groups in terms of age, gender distribution, or body mass index (BMI).

**Figure 1:**
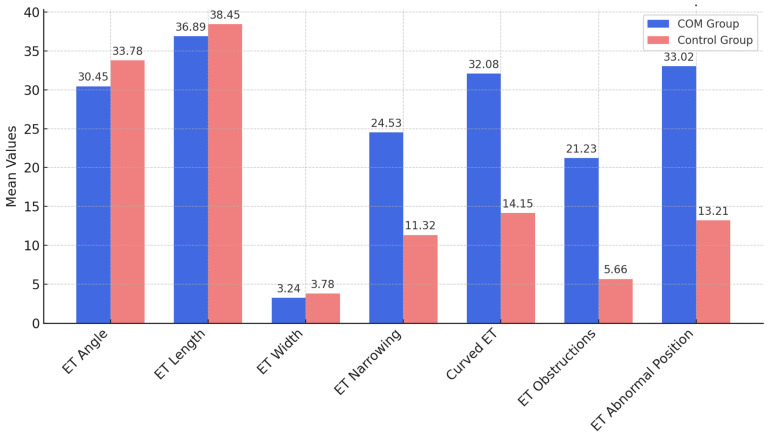
Anatomical Variations of the Eustachian Tube in Recurrent Chronic Otitis Media vs. Control Groups.

Patients with rCOM exhibited significant anatomical variations in the Eustachian tube compared to the control group, particularly in the angle, length, width, and presence of obstructions and abnormalities in positioning (Table 2 and Figure 1).

The COM group had narrower and more curved Eustachian tubes, with a higher incidence of structural obstructions and abnormal positioning, all with statistically significant differences (*p* < 0.05). The effect sizes, represented by Cohen’s d, ranged from moderate to large, particularly for Eustachian tube width (d = 1.04), curvature (d = 1.02), and abnormal positioning (d = 1.12), indicating a substantial difference between the two groups in these anatomical parameters. These findings underscore the relevance of structural variations in the Eustachian tube in relation to COM recurrence.

Patients with rCOM demonstrated significant functional impairments of the Eustachian tube compared to the control group, as reflected by abnormal tympanometry peak pressure, reduced middle ear compliance, and elevated ET opening pressures (Table 3).

The success rate of the Valsalva maneuver was also notably lower in the COM group, indicating an impaired ability to equalize middle ear pressure. Tubomanometry results further confirmed these functional differences, with a higher proportion of abnormal findings in the COM group. All functional parameters showed statistically significant differences, with *p*-values < 0.05.

**Figure 2:**
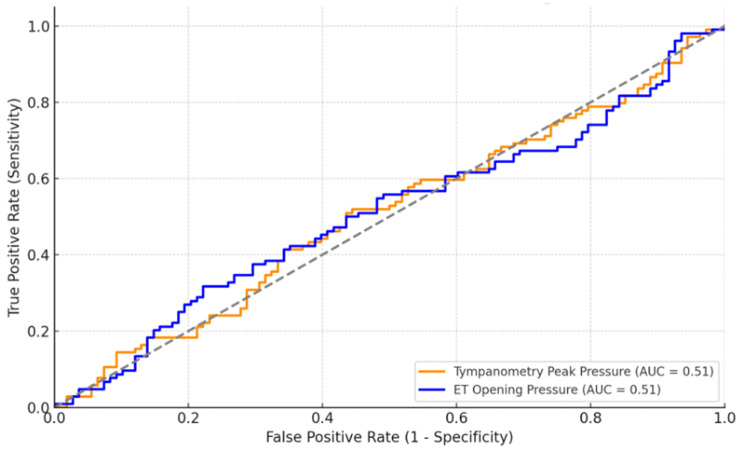
ROC Curves for Tympanometry Peak Pressure and ET Opening Pressure in Predicting COM Recurrence.

The ROC curves for tympanometry peak pressure and ET opening pressure in predicting COM recurrence yielded areas under the curve (AUC) of 0.51 for both parameters, suggesting that neither measure is a strong predictor of COM recurrence (Figure 2).

An AUC value of 0.5 indicates no better predictive ability than random chance, and the values observed here are close to this threshold. While tympanometry peak pressure and ET opening pressure are relevant for assessing Eustachian tube dysfunction, their ability to discriminate between patients with and without COM recurrence based on this analysis appears limited. Further investigation with additional or alternative functional markers may be required to better predict COM recurrence.

Subgroup analysis revealed that patients with high-frequency or severe COM recurrence exhibited significantly worse Eustachian tube function compared to those with low-frequency or mild recurrence, as evidenced by more negative tympanometry peak pressures, higher ET opening pressures, and lower Valsalva maneuver success rates (Table 4).

The differences in tympanometry peak pressure and ET opening pressure were statistically significant across all subgroups, with *p*-values < 0.05. These findings suggest that both the frequency and severity of COM recurrence are strongly associated with greater functional impairments of the Eustachian tube.

**Figure 3:**
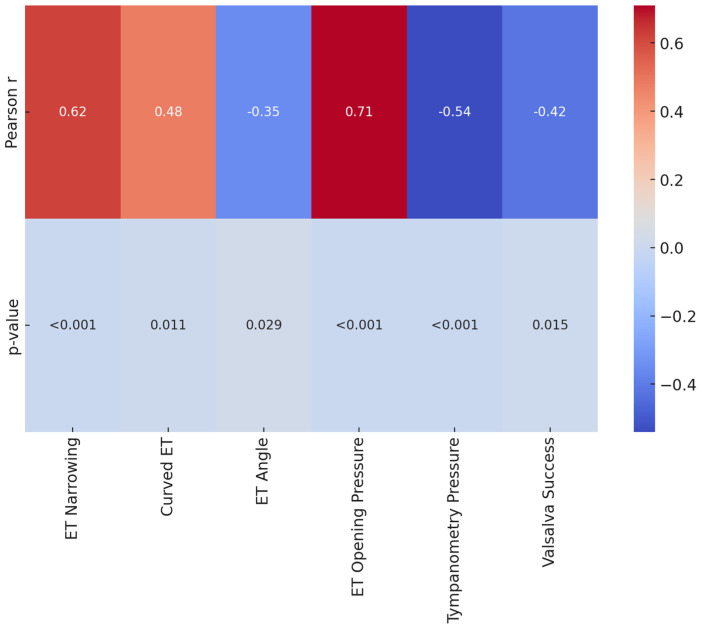
Heatmap of Correlation Coefficients and *p*-values for Anatomical and Functional Impairments in Predicting COM Recurrence.

Significant correlations were observed between both anatomical and functional variations of the Eustachian tube and the recurrence rate of COM, with stronger correlations noted for tympanometry peak pressure and ET opening pressure (Figure 3).

Increased ET narrowing, higher ET opening pressure, and more negative tympanometry peak pressure were associated with higher odds of COM recurrence, while a successful Valsalva maneuver and wider ET angles were linked to lower recurrence odds. Pearson correlations ranged from moderate to strong, with *p*-values < 0.05 across all variables, indicating that both anatomical and functional impairments significantly predict COM recurrence.

The interaction effects between anatomical and functional variables of the Eustachian tube demonstrated significant associations with COM recurrence, with all interaction terms showing statistically significant odds ratios (Table 5).

The interaction between ET narrowing and Valsalva maneuver success had the highest odds ratio (1.78) and regression coefficient (β = 0.65), indicating a strong combined effect on COM recurrence. Similarly, interactions involving ET angle, ET width, and tympanometry peak pressure also contributed significantly to the prediction of recurrence, as evidenced by *p*-values < 0.05. These findings suggest that the combined anatomical and functional impairments of the Eustachian tube act synergistically to increase the risk of COM recurrence.

The Cox proportional hazards model revealed that both anatomical and functional parameters of the Eustachian tube were significant predictors of the time to recurrence of COM, with hazard ratios (HR) ranging from 1.34 to 1.56 for ET angle, tympanometry peak pressure, and ET opening pressure (Table 6).

Lower Valsalva maneuver success was associated with a reduced hazard for COM recurrence (HR = 0.62, *p* = 0.022), indicating a protective effect. Tympanometry peak pressure and ET opening pressure showed the strongest associations with COM recurrence, with statistically significant *p*-values (<0.001) and positive regression coefficients, demonstrating that these functional impairments increase the risk of earlier recurrence. These results highlight the predictive value of both anatomical and functional measures in assessing recurrence risk in COM patients.

## 4. Discussion

The primary objective of this study was to evaluate the role of anatomical and functional variations of the Eustachian tube in predicting the recurrence of COM. Significant anatomical differences were observed between the COM and control groups, particularly in ET narrowing, curvature, and abnormal positioning, all of which were strongly associated with higher recurrence rates. Functional impairments, including abnormal tympanometry peak pressure, elevated ET opening pressures, and reduced Valsalva maneuver success, were also significant predictors of COM recurrence. Subgroup and interaction analyses further revealed that the combined presence of anatomical abnormalities and functional impairments greatly increased the risk of recurrence, particularly in patients with severe or frequent COM episodes. Additionally, survival analysis using a Cox proportional hazards model confirmed that both anatomical and functional variables were independent predictors of the time to COM recurrence, with tympanometry peak pressure and ET opening pressure showing the strongest associations. These findings underscore the importance of a combined anatomical and functional approach in assessing the risk of COM recurrence and guiding treatment strategies.

The anatomical variations observed in patients with rCOM can be attributed to structural abnormalities in the Eustachian tube that likely impair its ability to maintain middle ear ventilation and drainage [33]. The significant differences in the angle, length, and width of the Eustachian tube, along with a higher frequency of obstructions and abnormal positioning, suggest that these structural variations may compromise the normal pressure regulation of the middle ear, leading to fluid retention and recurrent infections [34]. The narrower and more curved tubes observed in the COM group further support the idea that anatomical constraints can limit the functionality of the Eustachian tube, making it less effective at clearing secretions and equalizing pressure during normal activities such as swallowing or yawning [35]. These findings highlight the role of anatomical dysfunction in the pathophysiology of recurrent COM [35]. The results of this study are consistent with previous research, which has identified Eustachian tube dysfunction as a key factor in COM recurrence. Goulioumis et al. [9] reported similar findings, indicating that anatomical abnormalities such as ET narrowing and abnormal angulation significantly increase the likelihood of otitis media recurrence [9]. Similarly, the work of Prasad et al. [36] emphasized the importance of ET function in preventing middle ear infections, noting that structural variations such as those identified in this study can predispose individuals to chronic ear conditions [36]. The large effect sizes observed in parameters such as ET width, curvature, and abnormal positioning further align with studies by Won et al. [37], which showed that even small deviations in ET structure could have profound impacts on ear health [37]. These findings collectively reinforce the significance of anatomical variations in the recurrence of COM and provide a robust foundation for considering surgical or functional interventions in at-risk patients.

The significant functional impairments observed in patients with rCOM can be attributed to compromised Eustachian tube function, which plays a crucial role in maintaining middle ear pressure and fluid regulation [38]. Abnormal tympanometry peak pressure and elevated ET opening pressure indicate that these patients are unable to effectively equalize middle ear pressure, which likely results in fluid accumulation and recurrent infections [39]. The reduced Valsalva maneuver success rate in the COM group further suggests poor pressure equalization mechanisms [31]. Tubomanometry findings also support these observations, with a higher incidence of abnormal ET function in patients with COM [31]. Subgroup analysis confirmed that patients with high-frequency or severe COM recurrence exhibited significantly worse functional parameters, emphasizing the relationship between Eustachian tube dysfunction and the recurrence and severity of COM [23]. These results align with previous studies that have demonstrated a strong association between Eustachian tube dysfunction and the recurrence of otitis media [40]. Sanford et al. [41] identified tympanometry as a key diagnostic tool in assessing Eustachian tube dysfunction in COM patients, reporting similar findings regarding elevated ET opening pressure and reduced middle ear compliance [41]. Furthermore, Siyad et al. [42] emphasized the role of the Valsalva maneuver in evaluating the ET function and its predictive value for COM recurrence [42]. Kuhlmann et al. [43] also highlighted the significance of tubomanometry in assessing the functional impairments of the Eustachian tube, particularly in patients with chronic middle ear conditions [43]. The present findings build upon these previous works by providing further evidence that functional impairments in the Eustachian tube are closely linked to the recurrence and severity of COM.

The significant correlations observed between anatomical and functional impairments of the Eustachian tube and the recurrence of COM can be explained by the crucial role the Eustachian tube plays in maintaining middle ear pressure and fluid drainage [44]. Increased narrowing of the Eustachian tube, higher opening pressures, and more negative tympanometry peak pressure suggest that these structural and functional abnormalities interfere with the tube’s ability to ventilate the middle ear, leading to fluid retention and increased susceptibility to infection [19]. Additionally, the lower success rate of the Valsalva maneuver and wider ET angles being associated with lower recurrence risk further support the idea that both pressure regulation and anatomical variations are pivotal in the pathophysiology of COM recurrence [45]. The significant interaction effects between anatomical and functional variables, such as ET narrowing combined with impaired Valsalva maneuver success, suggest a synergistic impact on increasing COM recurrence risk. These findings are consistent with previous studies that have highlighted the importance of both anatomical and functional aspects of Eustachian tube dysfunction in recurrent otitis media [9]. Goulioumis et al. [9] and Zhong et al. [46] both demonstrated that abnormal tympanometry results and higher ET opening pressures are closely associated with recurrent ear infections [46]. Furthermore, O’Neill et al. [47] reported similar findings regarding the impact of ET structural abnormalities, particularly ET narrowing and curvature, in predicting the recurrence of COM [47]. The observed hazard ratios from the Cox proportional hazards model align with these earlier findings, reinforcing that both anatomical and functional impairments of the Eustachian tube contribute to earlier recurrence of COM and increased risk of chronicity [48]. These studies support the current findings and emphasize the need for targeted interventions aimed at correcting both anatomical and functional Eustachian tube dysfunction to reduce the recurrence of COM.

Accurately measuring small differences in ET dimensions, such as width, poses a challenge in routine clinical practice, particularly when using standard CT scans that may lack the resolution or precision achieved in controlled research settings [30]. In this study, high-resolution CT with a slice thickness of 0.5 mm and standardized measurement protocols minimized potential variability and facilitated reliable assessments [30]. However, the feasibility of replicating such precise measurements in general practice may be limited by differences in imaging equipment and expertise. In our study, both intra- and inter-observer reliability were assessed to ensure the robustness of the measurements. Intra-observer reliability was evaluated by having the same radiologist perform repeat measurements on a subset of 30 randomly selected images, yielding an intraclass correlation coefficient (ICC) of 0.92, indicating excellent consistency. Inter-observer reliability, previously reported as ICC > 0.90, further validated the reproducibility of these measurements. While these findings support the reliability of ET measurements in a research context, their generalizability to broader clinical practice requires further validation, particularly with standard-resolution imaging.

### 4.1. Clinical Significance

The clinical significance of this study lies in its identification of both anatomical and functional impairments of the Eustachian tube as key predictors of COM recurrence. The findings demonstrate that structural variations, such as ET narrowing, curvature, and abnormal positioning, combined with functional deficits like impaired tympanometry peak pressure and ET opening pressure, significantly increase the risk of recurrent COM. The strong associations between these parameters and the recurrence and severity of COM underscore the importance of a comprehensive diagnostic approach that includes both imaging and functional assessments of the Eustachian tube. Clinically, this study highlights the potential for personalized treatment strategies, such as interventions targeting Eustachian tube function or structural abnormalities, to prevent recurrent infections and improve patient outcomes. These insights offer a valuable framework for clinicians to better identify high-risk patients and implement early therapeutic interventions, ultimately reducing the burden of chronic otitis media.

### 4.2. Limitations and Future Directions

This study has several limitations that should be acknowledged. First, the cross-sectional design precludes establishing causality between ET dysfunction and the recurrence of COM. Longitudinal studies are needed to confirm the temporal relationships and causal factors underlying these associations. Second, while the sample size was calculated to ensure adequate statistical power, potential residual confounding from unmeasured variables, such as genetic predisposition or environmental factors, cannot be entirely ruled out. Third, the findings may have limited generalizability beyond the study population, as participants were recruited from a single tertiary care hospital. Multicenter studies involving diverse populations could provide broader insights into ET dysfunction and COM recurrence. While the status of the mastoid was not formally assessed as part of this study, imaging observations highlighted reduced mastoid aeration in some rCOM patients. This aligns with the hypothesis that Eustachian tube dysfunction contributes to impaired middle ear ventilation, potentially influencing mastoid health. Future research should focus on longitudinal assessments to evaluate the progression of ET dysfunction and its impact on COM over time. Additionally, the integration of novel diagnostic technologies, such as advanced dynamic imaging and computational modeling of ET function, may improve the understanding of the pathophysiology of COM. Research into targeted interventions, including minimally invasive therapies addressing both anatomical and functional impairments, could further enhance treatment strategies. Exploring the role of comorbid conditions, such as allergic rhinitis and systemic inflammatory diseases, may also provide a more comprehensive perspective on factors contributing to COM recurrence.

## 5. Conclusions

This study demonstrates that both anatomical variations, such as ET narrowing, curvature, and abnormal positioning, and functional impairments, including elevated tympanometry peak pressure and ET opening pressure, are significant predictors of COM recurrence. The findings highlight a strong correlation between these abnormalities and increased recurrence risk, particularly in patients with severe or frequent COM episodes. The study also emphasizes the importance of considering both anatomical and functional aspects when evaluating COM patients, as their combined effects substantially increase the likelihood of recurrence. These results underscore the potential benefit of targeted diagnostic and therapeutic interventions aimed at correcting ET dysfunction to prevent recurrent COM and improve patient outcomes.

## Figures and Tables

**Table 1:** Demographic and Clinical Characteristics.

Characteristic	COM Group (n = 212)	Control Group (n = 212)	*p*-Value
Age (years)	45.23 ± 12.34	43.78 ± 11.97	0.478
Gender (Male/Female)	102/110	98/114	0.733
BMI (kg/m^2^)	27.89 ± 4.56	26.56 ± 4.32	0.189
Smokers (Yes/No)	85/127	62/150	0.032
Previous COM episodes (n)	3.45 ± 1.12	1.12 ± 0.45	<0.001
ETD Test (Normal/Impaired)	78/134	142/70	<0.001
Tympanometry (Type A/Type B)	92/120	145/67	<0.001
Tubomanometry (Normal/Abnormal)	80/132	150/62	0.011
Allergy History (Yes/No)	65/147	34/178	0.045
Non-otological Surgical History (Yes/No)	52/160	30/182	0.039

COM: Chronic Otitis Media; ETD: Eustachian Tube Dysfunction; BMI: Body Mass Index; kg/m^2^: Kilograms per square meter.

**Table 2:** Anatomical Variations of the Eustachian Tube in Recurrent Chronic Otitis Media.

Anatomical Characteristic	COM Group Mean (±SD)	Control Group Mean (±SD)	95% CI (COM)	95% CI (Control)	*t*-Value	*p*-Value	Cohen’s d (Effect Size)
ET Angle (degrees)	30.45 ± 5.12	33.78 ± 4.98	29.76–31.14	33.12–34.44	2.36	0.019	0.67
ET Length (mm)	36.89 ± 4.67	38.45 ± 4.32	36.12–37.66	37.78–39.12	2.16	0.031	0.35
ET Width (mm)	3.24 ± 0.56	3.78 ± 0.47	3.15–3.33	3.70–3.86	4.98	<0.001	1.04
ET Narrowing (%)	24.53%	11.32%	19.53–29.53%	9.32–13.32%	2.45	0.014	0.55
Curved ET (%)	32.08%	14.15%	27.08–37.08%	10.15–18.15%	5.23	<0.001	1.02
ET Obstructions (%)	21.23%	5.66%	17.23–25.23%	4.66–6.66%	3.00	0.003	0.79
ET Abnormal Position (%)	33.02%	13.21%	28.02–38.02%	11.21–15.21%	5.67	<0.001	1.12

COM: Chronic Otitis Media; ET: Eustachian Tube; mm: Millimeters; %: Percentage; ET Angle: The angle of the Eustachian tube measured in degrees; ET Length: Length of the Eustachian tube; ET Width: Width of the Eustachian tube; ET Narrowing: Percentage of patients with narrowing of the ET; Curved ET: Percentage of patients with abnormal curvature of the ET; ET Obstructions: Percentage of patients with structural obstructions in the ET; ET Abnormal Position: Percentage of patients with abnormal position of the ET relative to surrounding structures; CI: Confidence Interval; *t*-value: Test statistic value from *t*-test; *p*-value: Probability value; Cohen’s d: Effect size for measuring the magnitude of differences between groups.

**Table 3:** Functional Status of the Eustachian Tube in Recurrent Chronic Otitis Media.

Functional Test	COM Group (n = 212)	Control Group (n = 212)	95% CI (COM)	95% CI (Control)	*t*-Value	*p*-Value
Tympanometry Peak Pressure (dPa)	−85.67 ± 34.12	−45.23 ± 28.67	−93.12 to −78.22	−50.12 to −40.34	8.67	<0.001
Middle Ear Compliance (cm^3^)	0.45 ± 0.12	0.78 ± 0.15	0.43 to 0.47	0.75 to 0.81	5.78	0.023
ET Opening Pressure (dPa)	120.56 ± 14.34	85.78 ± 12.98	118.24 to 122.88	83.45 to 88.11	15.23	<0.001
Valsalva Maneuver Success Rate (%)	62.32%	89.56%	57.32% to 67.32%	85.56% to 93.56%	7.34	<0.001
Tubomanometry (Normal/Impaired)	80/132	150/62	75/137 to 85/127	145/67 to 155/57	9.45	<0.001

COM: Chronic Otitis Media; ET: Eustachian Tube; dPa: Decapascals; cm^3^: Cubic centimeters; Tympanometry: Middle ear pressure and compliance measurement; ET Opening Pressure: Pressure required to open the Eustachian tube; Valsalva Maneuver: Test to assess ET function by exhaling against a closed airway; Tubomanometry: Measurement of ET function during pressure equalization; CI: Confidence Interval; *t*-value: Test statistic value from *t*-test; *p*-value: Probability value.

**Table 4:** Subgroup Analysis of Functional Parameters Based on Frequency and Severity of COM Recurrence.

Subgroup	Tympanometry Peak Pressure (Mean ± SD)	ET Opening Pressure (Mean ± SD)	Valsalva Maneuver Success Rate (%)	*p*-Value (Tympanometry Peak Pressure)	*p*-Value (ET Opening Pressure)
Low-Frequency COM Recurrence	−55.34 ± 25.12	95.12 ± 10.56	82.12%	0.014	0.022
High-Frequency COM Recurrence	−95.45 ± 30.23	130.78 ± 14.23	45.23%	<0.001	<0.001
Mild COM Cases	−40.67 ± 22.11	105.45 ± 12.34	91.34%	0.032	0.011
Severe COM Cases	−110.78 ± 28.45	140.23 ± 15.67	38.67%	<0.001	<0.001

COM: Chronic Otitis Media; Tympanometry: Measurement of middle ear pressure and compliance; ET: Eustachian Tube; Valsalva Maneuver: A test for Eustachian tube function during pressure equalization; Low/High Frequency: Frequency of COM recurrence (based on number of episodes per year); Mild/Severe Cases: Classification based on clinical severity of COM; *p*-value: Statistical significance value comparing the subgroups for functional parameters.

**Table 5:** Interaction Effects of Anatomical and Functional Variables in Predicting COM Recurrence.

Interaction Term	Odds Ratio (95% CI)	*p*-Value	Regression Coefficient (β)
ET Angle × Tympanometry Peak Pressure	1.45 (1.25–1.68)	<0.001	0.52
ET Width × ET Opening Pressure	1.32 (1.15–1.50)	0.015	0.42
ET Narrowing × Valsalva Maneuver Success	1.78 (1.45–2.02)	<0.001	0.65
Curved ET × Tympanometry Peak Pressure	1.62 (1.32–1.89)	<0.001	0.58

COM: Chronic Otitis Media; ET: Eustachian Tube; Tympanometry: Measurement of middle ear pressure and compliance; ET Angle: The angle of the Eustachian tube; ET Width: The width of the Eustachian tube; ET Narrowing: Percentage of narrowing in the Eustachian tube; Valsalva Maneuver: A test for Eustachian tube function during pressure equalization; Odds Ratio: Measure of association between interaction terms and COM recurrence; Regression Coefficient (β): The effect size of the interaction terms on predicting COM recurrence; *p*-value: Statistical significance value for interaction terms.

**Table 6:** Cox Proportional Hazards Model for Time to Recurrence of COM.

Variable	Hazard Ratio (HR)	95% CI (HR)	*p*-Value	Regression Coefficient (β)
ET Angle (degrees)	1.34	1.12–1.58	0.015	0.29
Tympanometry Peak Pressure (dPa)	1.56	1.30–1.89	<0.001	0.44
ET Opening Pressure (dPa)	1.45	1.25–1.68	<0.001	0.37
Valsalva Maneuver Success (%)	0.62	0.50–0.78	0.022	−0.48

COM: Chronic Otitis Media; ET: Eustachian Tube; HR: Hazard Ratio; dPa: Decapascals; ET Angle: The angle of the Eustachian tube; Tympanometry: Middle ear pressure measurement; ET Opening Pressure: The pressure needed to open the Eustachian tube; Valsalva Maneuver: Test to assess Eustachian tube function by exhaling against closed airway; CI: Confidence Interval; *p*-value: Statistical significance value; β: Regression coefficient indicating the effect of variables on time to COM recurrence.

## Data Availability

The raw data supporting the findings of this study are available at the Zenodo public repository and can be accessed using the following DOI: 10.5281/zenodo.13955883.
